# Variables Affecting Thigh Girth Measurement and Observer Reliability in Dogs

**DOI:** 10.3389/fvets.2018.00203

**Published:** 2018-08-30

**Authors:** Daniel A. McCarthy, Darryl L. Millis, David Levine, Joseph P. Weigel

**Affiliations:** ^1^Department of Small Animal Clinical Sciences, University of Tennessee College of Veterinary Medicine, Knoxville, TN, United States; ^2^Department of Physical Therapy, The University of Tennessee at Chattanooga, Chattanooga, TN, United States

**Keywords:** thigh circumference, muscle atrophy, muscle atrophy post-cranial cruciate ligament transection, dog thigh girth, measure thigh girth

## Abstract

**Objective:** The purpose of the study reported here was to describe variables affecting thigh girth measurements preoperatively and 2 weeks after surgical stabilization of the stifle and to examine inter- and intra-observer reliability.

**Animals:** Ten hound-type dogs with experimental, unilateral, cranial cruciate ligament transection, and surgical stabilization.

**Procedures:** Dogs were placed in lateral recumbency for measurements of thigh circumference after the stifle was placed in flexed (F), estimated standing (S), and extended (E) positions. Measurements were made at 50 and 70% of thigh length (TL), with hair unclipped and then clipped prior to surgery, before and 2 weeks after cruciate ligament transection and stifle stabilization, and with and without sedation. A spring tension measuring tape was used to determine thigh girth that allowed a consistent amount of end-tension to be applied to the tape. All measurements were made by two blinded individuals in triplicate, data were recorded for each set of measurements and the mean of the three measurements for each condition was used for analysis.

**Results:** Thigh girth was significantly greater at the more proximal site of 50% TL (36.7 ± 2.6 cm) when compared to the 70% TL (31.7 ± 2.7 cm) (*P* = 0.001). Sedation did not significantly affect thigh girth at any stifle position at the 70% and 50% TL. Although there were no differences in thigh circumference between the flexion and standing positions at 50% TL (F 38.2 ± 2.8 cm, S 38.1 ± 2.9 cm) and 70% TL (F 33.6 ± 1.6 cm; S 33.6 ± 1.8 cm), full extension of the stifle resulted in significantly less thigh girth (50% TL 36.6 ± 2.6 cm, *P* = 0.006; 70% TL 31.7 ± 2.6 cm, *P* = 0.006). Significant decreases in thigh girth were seen after surgery in all limb positions at both measurement sites. The highest correlations between Observer 1 (OB1) and Observer 2 (OB2) with least differences in measurements were with the stifle in the extended position. Agreement between two observers using standard measuring technique was significant at both the 50% (OB1: 34.10 ± 2.93 cm, OB2: 34.08 ± 2.65 cm, *P* = 0.007, ICC = 0.984) and 70% (OB1: 29.89 ± 2.43 cm, OB2: 30.04 ± 2.30 cm, *P* = 0.004, ICC = 0.981) TL positions with the stifle placed in extended position.

**Conclusion and Clinical Importance:** Thigh girth measurement may be useful as an outcome measure when appropriate measuring technique is used. It is recommended that thigh girth be obtained at a distance of 70% thigh length, with the leg in an extended position while in lateral recumbency, and the dog relaxed or under sedation. Further studies should be performed in a variety of clinical situations.

## Introduction

Muscle atrophy commonly occurs following injury and surgery (1, 2). Assessment and improvement of medical, surgical, and physical rehabilitation treatments for various conditions depend on accurate, inexpensive, and reliable methods of measuring outcome parameters. In human medicine, thigh girth has been used as a reliable functional outcome objective measurement to evaluate progress and recovery (1, 3–7). The quadriceps muscle group comprises a majority of the cranial thigh susceptible to atrophy, and the biceps femoris, semitendinosus, and semimembranosus muscles comprise the caudal thigh muscles susceptible to atrophy. Their location allows sufficient access to palpate, measure, and evaluate the muscles under the proper conditions.

The quadriceps muscle group is particularly prone to atrophy secondary to decreased limb function from musculoskeletal injury. In human patients with anterior cruciate ligament tears, there was no significant atrophy of any other muscle except the quadriceps muscle group during muscle measurements (1). The human quadriceps muscles of the anterior cruciate ligament-deficient limb were significantly weaker (average 25%) than those of the uninjured side; the total quadriceps, vastus lateralis, and vastus intermedius volume and cross-sectional area were significantly smaller in the anterior cruciate ligament-deficient limb (1). Therapy directed toward improving muscle strength and mass is likely to improve patient function and overall outcome.

Thigh girth measurements in humans have been considered both reliable and repeatable as long as a standardized measurement protocol is used (6, 7). A standard protocol is vital in decreasing observer variability. A recent study in dogs used a laser device to ensure precise location of landmarks to standardize the location of thigh girth measurement (8). Although these dogs were placed in relative standing angles, alterations in the flexion or extension angle of the stifle joint may have altered thigh girth, resulting in a low agreement of measurements between observers. More consistent positioning of the limb and joints may have improved agreement of measurements by different observers.

Thigh girth measurements have been used to assess progress following surgical management of cranial cruciate ligament repair and canine total knee arthroplasty and its use has been explored for other purposes (9–11). One study found that thigh circumference was statistically decreased compared to the unaffected contralateral limb in dogs 1 and 5 years after stifle stabilization surgery for naturally occurring cranial cruciate ligament disease (12).

A simple, repeatable method of determining thigh girth is necessary to assess changes in thigh girth as a non-invasive, clinically applicable method of estimating muscle mass. Although thigh girth measurement has been studied in veterinary patients, differences between observers have been problematic (8, 13). Standard technique and limb positioning may result in improved agreement among observers. In addition, measurement of thigh girth should be simple, inexpensive, reproducible, and relatively quick. Variables that may affect thigh girth include location of the measurement on the thigh, angle of limb in flexion or extension, whether the hair is clipped or not, determination of girth in awake or sedated dogs, and reproducibility between evaluators.

To the author's knowledge, no studies have evaluated the use of thigh girth measurement in dogs prior to and following surgery of the stifle under various positions and conditions. The purpose of the study reported here was to evaluate the effect of stifle joint position, clipping, sedation, and different evaluators on thigh girth measurements at two different locations before and 2 weeks after transection of a cranial cruciate ligament and immediate stabilization of the stifle. We hypothesized that extension of the stifle, measurement of the distal thigh, clipping the hair, and sedation of dogs would result in lower thigh girth as compared with flexion of the stifle, measurement of the proximal thigh, unclipped limbs, and non-sedated dogs. We also hypothesized there would be acceptable intra- and inter-observer repeatability when a standard measurement technique was used.

## Materials and methods

Thigh girth was measured using 10 young, adult male and female mixed-breed hound-type dogs. All dogs were determined to be healthy on the basis of physical examinations, complete blood count (CBC), and serum chemistry profiles. The dogs were part of another study in which a unilateral cranial cruciate ligament was transected and the stifle immediately stabilized using an extracapsular technique. This study was carried out in accordance with the recommendations of University of Tennessee's Institutional Animal Care and Use Committee. The protocol was approved by the University of Tennessee's Institutional Animal Care and Use Committee prior to commencement.

**Figure 1:**
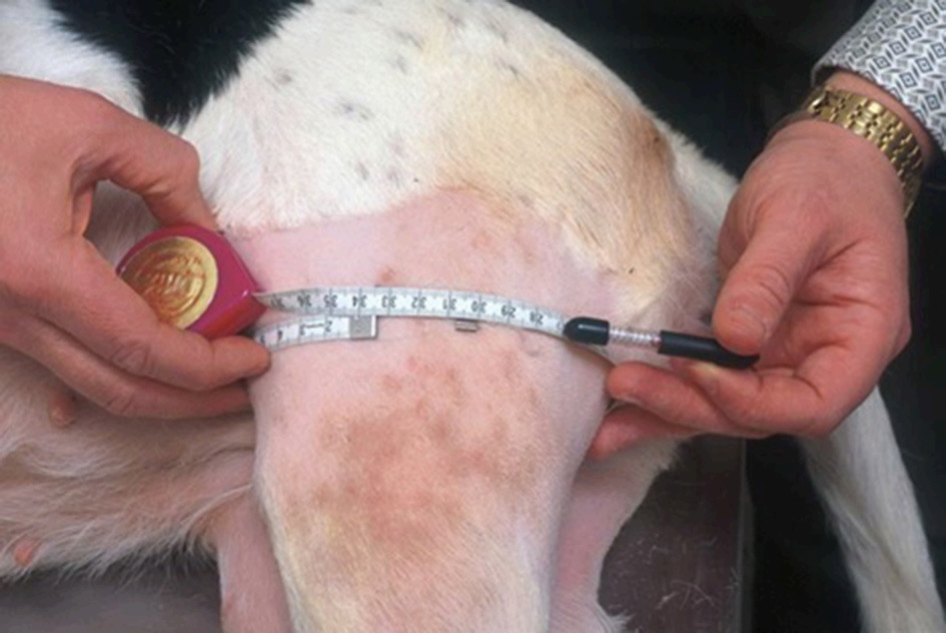
A Gulick II measuring tape was used to determine thigh girth. The tape was placed around the limb with a consistent amount of end-tension placed on the tissues minimizing differences in the amount of tension after the tape was pulled. The tape measure was pulled taut until one of the red balls was completely exposed (4 oz. of end tension).

A Gulick II measuring tape^1^ was used to determine thigh girth. This instrument allowed the tape to be placed around the limb with a consistent amount of end-tension placed on the tissues, thereby, minimizing differences in the amount of tension after the tape was pulled taut. The tape measure was tensioned until one of the red balls was completely exposed (4 oz. [133.4 g] of end tension) (Figure 1). Care was taken to ensure that the tape did not slip distally during tensioning. Dogs were placed in lateral recumbency for measurements. All measurements were made by two individuals after a training period. Both evaluators practiced the technique, including palpation of anatomic landmarks, on at least 20 other dogs prior to study start.

To make the measurements, Observer 1 entered the room, measured thigh length and marked the site of interest. An assistant placed the limb in the position to be measured (full flexion, full extension or an estimated standing angle) while the observer placed the tape around the limb and pulled the tape until the desired amount of tension was reached. The observer held the tape in place without looking at the tape measure value while the assistant recorded the value. The measuring tape was then completely removed, and the limb was repositioned prior to placing the limb in the desired stifle angle to make the second and third measurements in the same fashion with minimal time lapse between each measurement. A total of 18 measurements were made for each observer. Following data collection, Observer 1 exited the room, Observer 2 entered the room and performed the measurements in a similar fashion. The means of each set of measurements (flexion, extension, and estimated standing angle) at both 50 and 70% thigh length were used for analysis.

### Effect of different measuring locations

**Figure 2:**
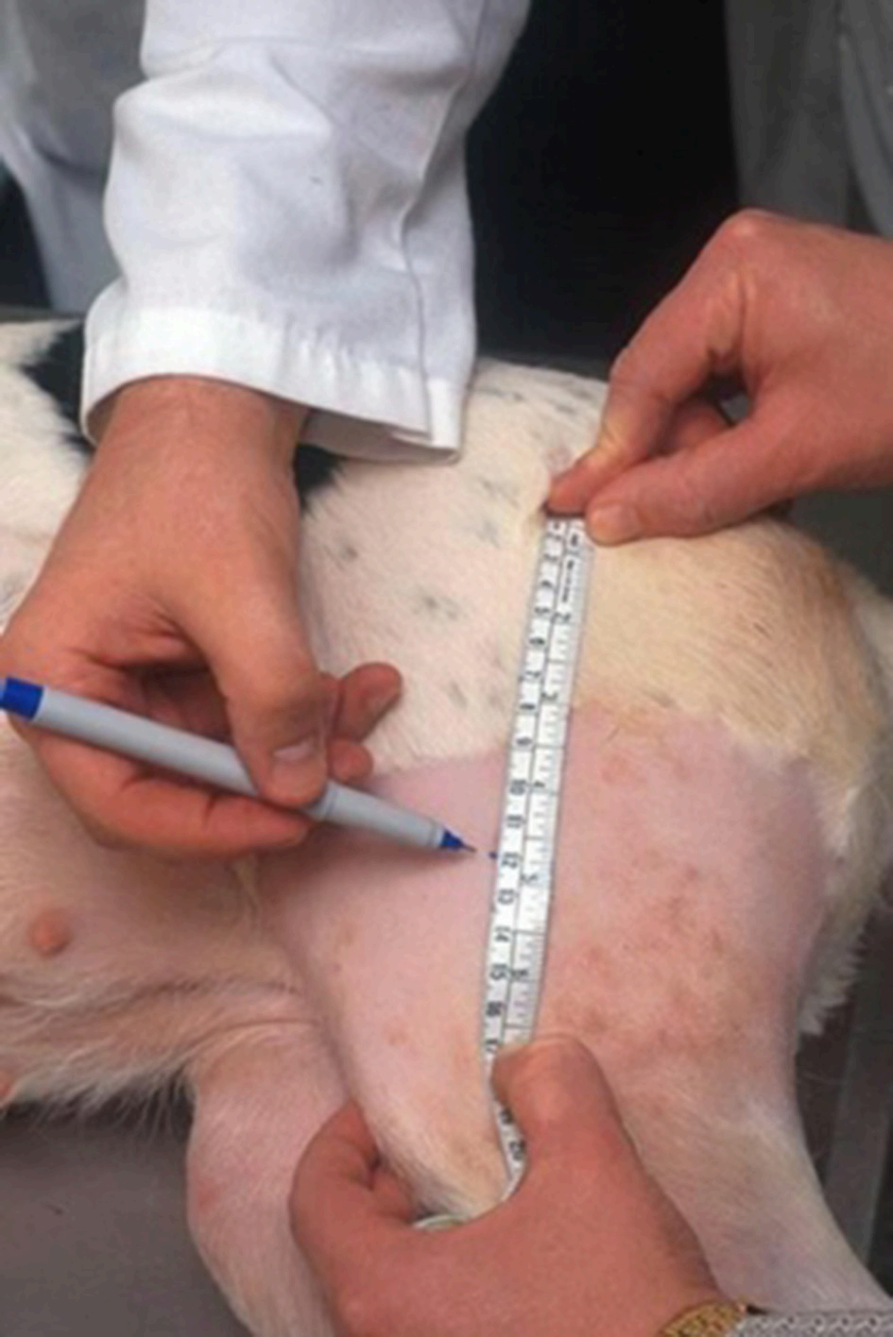
Thigh length (TL) was determined by measuring from the proximal tip of the greater trochanter to the distal aspect of the lateral fabella. A surgical marking pen was used to mark points equal to 50 and 70% of the thigh length, as measured from the tip of the greater trochanter.

Thigh length (TL) was determined by measuring from the proximal tip of the greater trochanter to the distal aspect of the lateral fabella (Figure 2). A surgical marking pen was used to mark points equal to 50 and 70% of the thigh length, as measured from the tip of the greater trochanter. Thigh girth was determined by placing the tape measure around the thigh at these points, ensuring the tape was perpendicular to the femur. To maintain blinding for OB2, OB1 made several marks to mask the actual site of measurement, and OB2 used a different color pen to make marks.

### Effect of clipping

To determine the contribution of hair on thigh girth, measurements were made on limbs of awake dogs prior to clipping and after clipping prior to surgery with the stifle at an estimated standing angle.

### Effect of stifle position

The effect of stifle position on thigh girth was determined by taking measurements with the stifle fully flexed, fully extended, and at an estimated functional standing angle. To estimate the standing angle, we attempted to create a floor surface by placing a hand under a paw, and placing the pelvic limb in the estimated standing angle with the tuber calcis and metatarsals placed perpendicular to the simulated floor surface. The hip was maintained in a functional standing angle (approximately 95–110°), and the tarsus was allowed to move passively while positioning the stifle.

### Effect of sedation

To determine the effects of sedation on muscle tension, measurements were made on dogs which were fully awake and after pre-medication with acepromazine^2^ (0.11 mg/kg, intramuscular).

### Effect of surgery

Thigh girth was measured before transection of the cranial cruciate ligament and stifle stabilization and 2 weeks after surgery to determine the changes after surgery. No special exercise or rehabilitation protocol was instituted after surgery.

### Intra-observer and inter-observer variability

Intra-observer and inter-observer variability were evaluated using intra- and interclass correlation coefficients. To determine the intra-observer variability, the same observer performed three measurements for each stifle position, at the 50 and 70% thigh lengths. To determine inter-observer variability of thigh girth measurements, the same measurements were made by two independent evaluators (OB1 and OB2), who were unaware of the other investigator's findings.

### Statistics

An *a priori* power analysis with a power of 0.80 and a *P*-value < 0.05 was performed using pilot data from three dogs to determine the number of subjects needed to detect differences between measurements collected by individual observers (intra-observer reliability) and different observers (inter-observer reliability). Analysis indicated that the sample size needed was three subjects for both intra- and inter-observer reliability.

Means of the three measurements were compared using paired *t*-test where appropriate. A repeated measures ANOVA was used to compare group means when more than two groups were being compared. If overall group differences were apparent, differences between groups were determined using the LSD procedure. The intraclass and interclass correlation coefficient (ICC) test was performed to compare the reliability and continuity between both inter- and intra-observer variability. The standard deviations of the individual measurements were all extremely small allowing us to average them for analyses. Statistical significance was established at *P* < 0.05.

## Results

**Figure 3:**
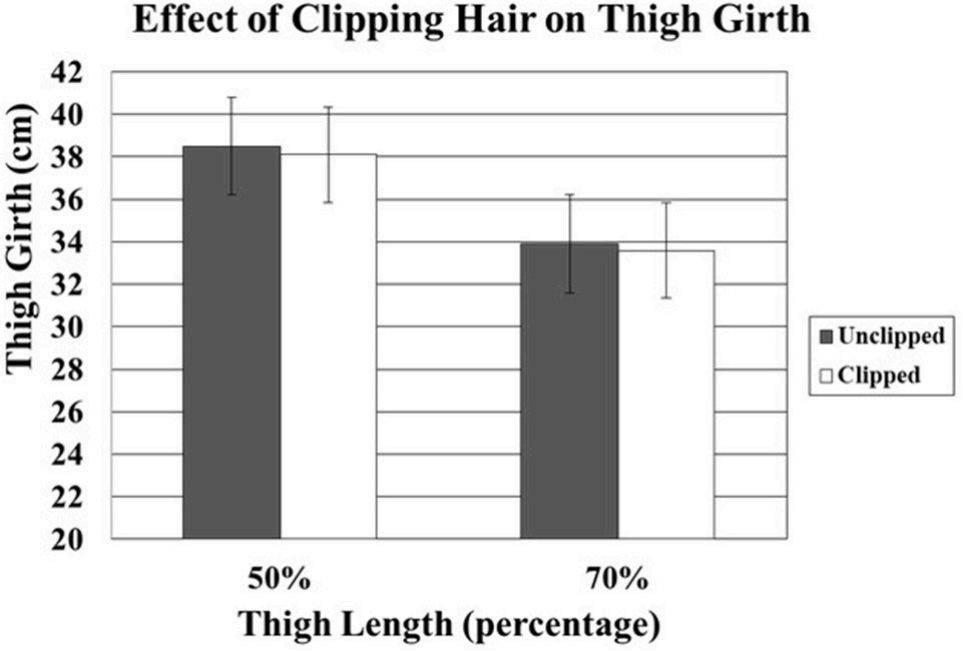
Comparison of effect of unclipped and clipped hair at 50% and 70% thigh circumferences. Although clipping the hair did not significantly affect thigh girth, mean thigh girth was 7 and 3 mm less in clipped limbs at the 50% and 70% thigh length locations, respectively.

**Figure 4:**
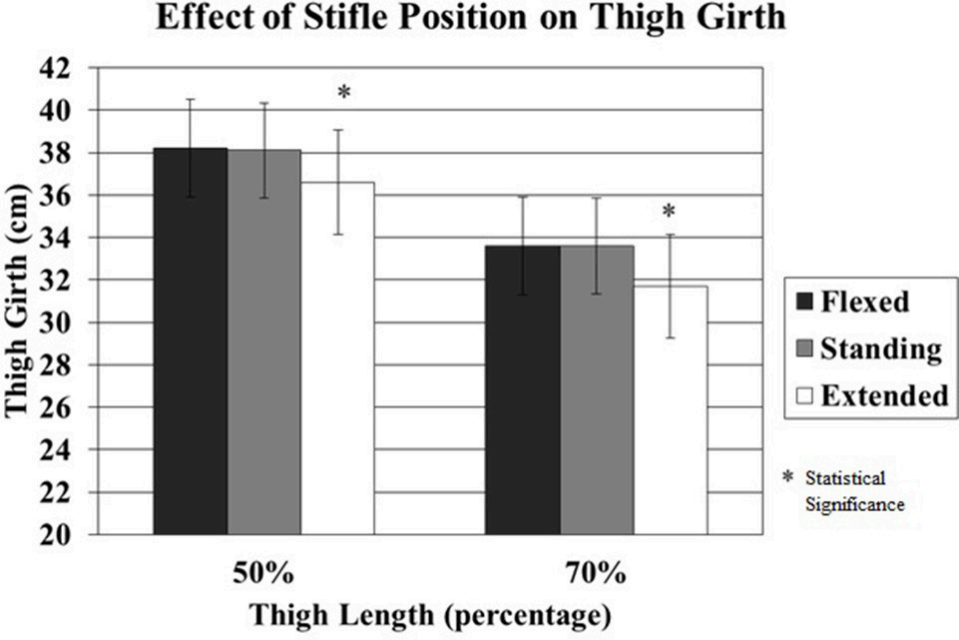
The effect of stifle position on thigh girth measured at 50% and 70% thigh length when the stifle was flexed, standing, and extended. Although there was little difference between flexion of the stifle and placement of the limb in a functional standing position, full extension of the stifle resulted in significantly less thigh girth.

A total of 1,200 measurements were performed by OB1 and OB2. Thigh girth was significantly greater at the more proximal 50% TL when compared to the 70% TL at all positions (Table 1). Although clipping the hair did not significantly affect thigh girth, mean thigh girth was 7 and 3 mm less in clipped limbs at the 50 and 70% TL locations, respectively, compared to the unclipped limbs (Tables 2, 3, Figure 3). Position of the stifle joint also affected measurements. Although there was little difference between flexion of the stifle and placement of the limb in an estimated functional standing position, full extension of the stifle resulted in significantly less thigh girth (Table 4, Figure 4).

**Table 1:** Comparison of thigh circumference (+/– SD) measured at 50% TL and 70% TL at three different stifle positions prior to sedation, clipping, and surgery.

	**50% TL**	**70% TL**	***P*-Value**
Flexion	38.2+/−2.8	33.6+/−1.6	0.001
Standing	38.1+/−2.9	33.6+/−1.8	0.002
Extension	36.6 +/−2.6	31.7+/−2.7	0.001

*All measurements are reported in centimeters*.

**Table 2:** Different conditions affecting thigh circumference measurements (+/–SD) at 50% TL at three different stifle positions.

**50% TL**	**Limb position pre-sx mean ± *SD***	**Post-sx mean ±*SD***	**Pre-clip mean ±*SD***	**Post-clip mean ±*SD***	**Pre-sedation mean ±*SD***	**Post-sedation mean ±*SD***
Flexion	38.2+/−2.8	34.7+/−3 (*P* = 0.0001)	-	-	34.7 +/−3	33.4+/−2.4 (*P* = 0.07)
Standing	38.1+/−2.9	34.3+/−2.5 (*P* = 0.0001)	38.8+/−2.7	38.1 +/−3.1 (*P* = 0.27)	34.3+/−2.5	34+/−2.7 (*P* = 0.32)
Extension	36.6 +/−2.6	33.2+/−2.2 (*P* = 0.00004)	-	-	33.2+/−2.2	32.8+/−2.4 (*P* = 0.14)

*Thigh girth was decreased at the stifle extension position relative to other stifle positions and in post-surgery flexion, extension, and estimated standing stifle positions. Pre-clip and post-clip measurements were only taken in the standing stifle position. All measurements are reported in centimeters*.

*sx, Surgery; SD, Standard Deviation*.

**Table 3:** Different conditions affecting thigh circumference measurements (+/– *SD*) at 70% TL at three different stifle positions.

**70% TL**	**Limb position pre-sx mean ±*SD***	**Post-sx mean ±*SD***	**Pre-clip mean ±*SD***	**Post-clip mean ±*SD***	**Pre-sedation mean ±*SD***	**Post-sedation mean ±*SD***
Flexion	33.6+/−1.6	31+/−2.1 (*P* = 0.00007)	-	-	31+/−2.1	29.8+/−1.9 (*P* = 0.07)
Standing	33.6+/−1.8	30.2+/−2.3 (*P* = 0.0002)	33.9 +/−2.6	33.6+/−1.8 (*P* = 0.27)	30.2 +/−2.3	30.1+/−2.4 (*P* = 0.42)
Extension	31.7+/−2.7 (*P* = 0.0001)	29.2+/−2 (*P* = 0.003)	-	-	29.1 +/−2	29.2+/−1.9 (*P* = 0.28)

Thigh girth was decreased at the stifle extension position relative to other stifle positions and in post-surgery flexion extension, and standing positions. Pre-clip and post-clip measurements were taken in the standing stifle position only.

*sx, Surgery; SD, Standard Deviation*.

**Table 4:** Comparison of thigh girth at 50 and 70% thigh lengths with the limb in an extended, standing, and flexed angle.

**Measurement position**	**OB1 mean ±*SD***	**OB2 mean ±*SD***	**ICC (Inter-rater class correlation with 95% CI)**	**ICC OB1 (Intra-rater class correlation with 95% CI)**	**ICC OB2 (Intra-rater class correlation with 95% CI)**
70% Extended	29.89 ± 2.43	30.04 ± 2.30	0.981 (955.−0.992)	0.993 (0.975–0.995)	0.994 (0.978–0.996)
70% Standing	31.74 ± 2.61	32.08 ± 2.33	0.972 (0.944–0.986)	0.989 (0.974–0.996)	0.991 (0.974–0.996)
70% Flexed	31.43 ± 2.45	31.10 ± 2.38	0.973 (0.942–0.989)	0.987 (968.−0.994)	0.992 (0.984–0.996)
50% Extended	34.10 ± 2.93	34.08 ± 2.65	0.984 (966.−0.994)	0.986 (0.966–0.994)	0.984 (0.964–0.992)
50% Standing	35.38 ± 3.24	35.04 ± 2.71	0.963 (0.945–0.989)	0.966 (0.921–0.986)	0.979 (0.958–0.989)
50% Flexed	35.36 ± 3.35	35.01 ± 2.96	0.959 (0.932–0.978)	0.964 (0.931–0.986)	0.972 (0.952–0.982)

*Highest inter- and intra-class correlations (ICC) between observers were found when the thigh girth was measured at the 50 and 70% with the stifle in an extended position. Measurements were made in centimeters. (SD, Standard Deviation; CI, Confidence Interval, P < 0.01 for all ICC values)*.

**Figure 5:**
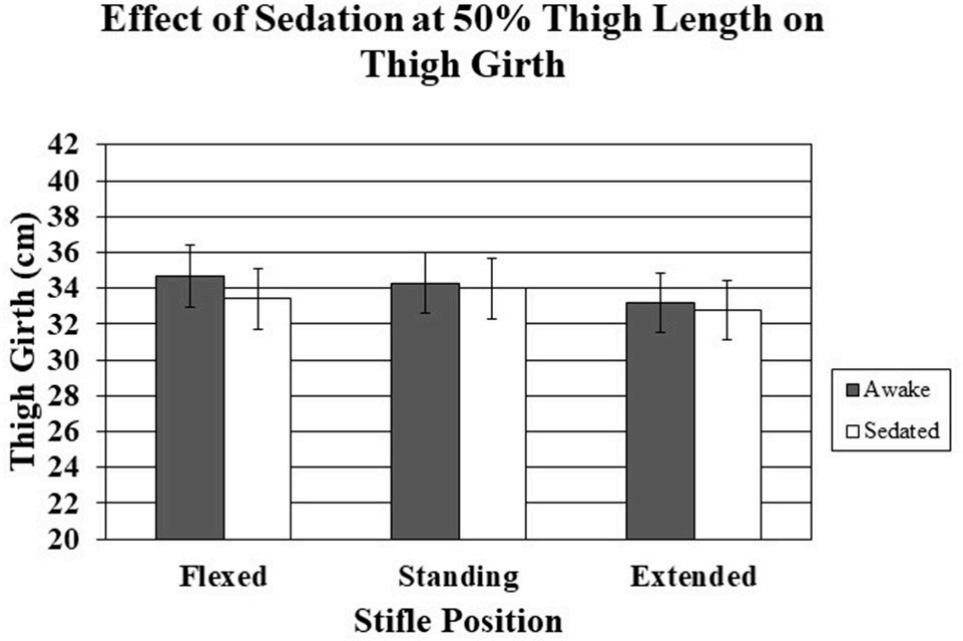
The effect of sedation on thigh girth measured at 50% thigh length when the stifle was flexed, standing, and extended. There were no significant differences.

**Figure 6:**
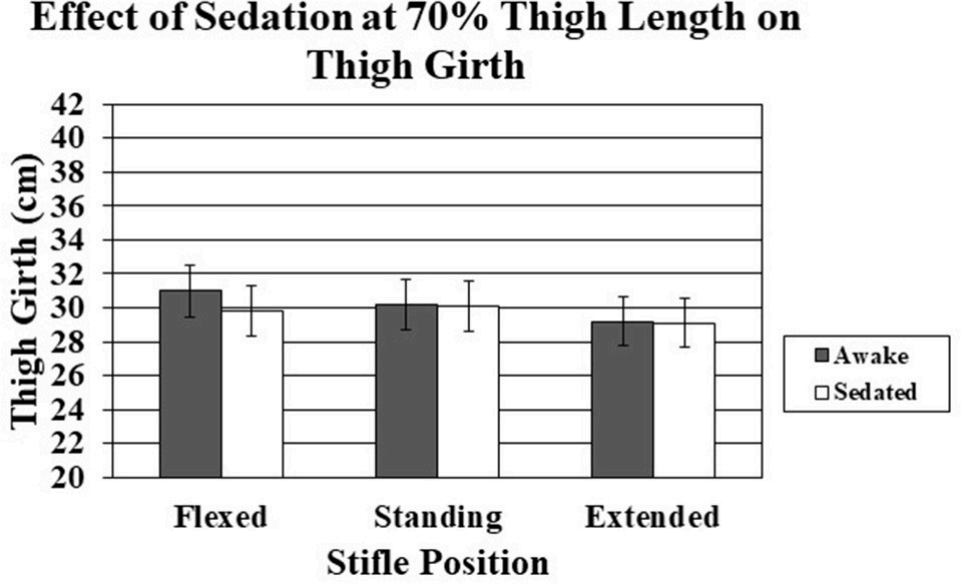
The effect of sedation on thigh girth measured at 70% thigh length when the stifle was flexed, standing, and extended. There were no statistical significant differences between sedated and non-sedated dogs.

Sedation had little effect on thigh girth in the standing and extended positions for both 50–70% thigh length sites (Tables 2, 3). However, there was a trend for decreased thigh girth of sedated dogs with the limb in the flexed position (*P* = 0.07) (Figures 5, 6).

**Figure 7:**
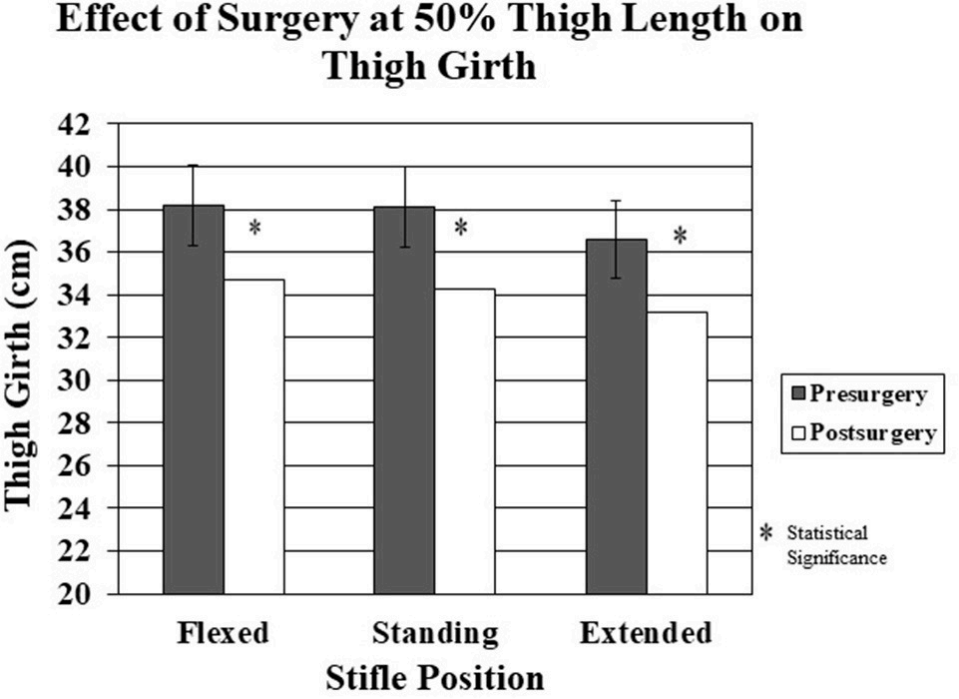
The effect of surgery on thigh girth at 50% thigh length pre-surgery vs. post-surgery in the flexed, standing, and extended stifle positions. There was a significantly decreased thigh girth for all stifle positions post-surgery.

**Figure 8:**
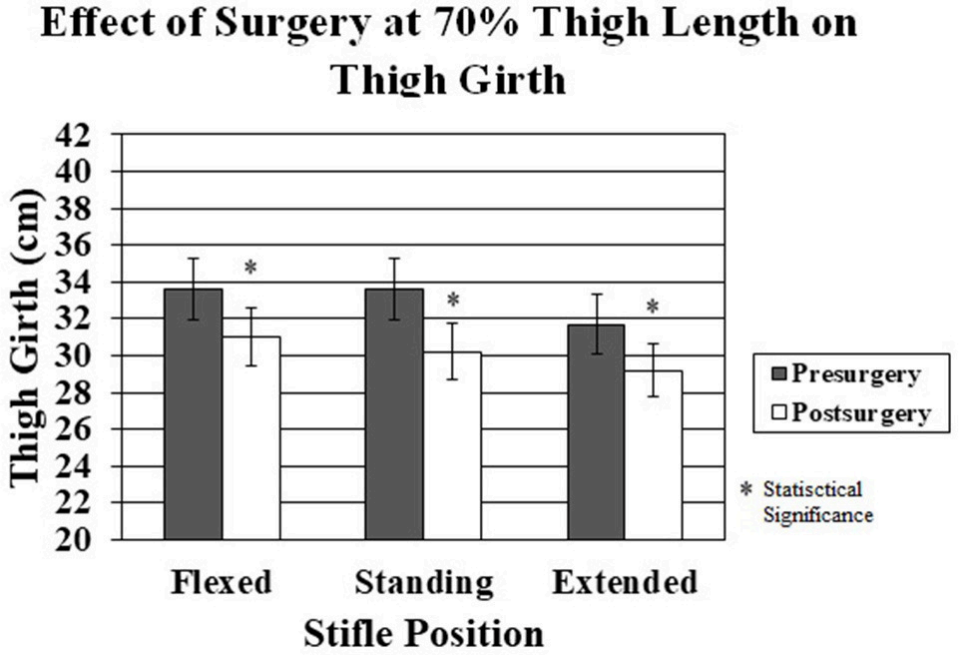
The effect of surgery on thigh girth at 70% thigh length pre-surgery vs. post-surgery in the flexed, standing, and extended stifle positions. There was a significant decrease in thigh girth in all stifle positions post-surgery.

Significant decreases in thigh girth were seen after surgery in all stifle positions at both measurement sites (Figures 7, 8). The decreases ranged from 2.5 cm for the 70% thigh length with the limb extended to 3.8 cm for the 50% thigh length with the limb in a standing position (Figures 7, 8).

Intraclass correlations were similar and highest with OB1 (0.993) and OB2 (0.994) for the thigh girth measurements made at 70% with the stifle in the extended position. Two observers using the standard measurement technique obtained similar results for thigh girth measurement. The highest interclass correlations (50% TL: ICC = 0.984, *P* = 0.007; 70% TL: ICC = 0.981, *P* = 0.004) between observers and the least differences between observers were for measurements made with the limb in the extended position (Table 4).

## Discussion

Thigh girth has long been an indirect method of assessing changes in muscle mass in people (14). Measurement of thigh girth is inexpensive, quick, and easily performed on clinical patients. Acceptable results depend on standard, repeatable methods of measuring thigh girth to obtain meaningful measurements. One factor in making reliable, repeatable measurements is the type of tape measure used. One study evaluating human thigh circumference measured by spring tape and optoelectronic volumetry revealed high reliability between the two methods (15). Another veterinary study compared the precision of four different types of tape measures used to measure dog thigh circumferences, and although significant differences were not found, the Gulick II tape measure had lower inter-observer and intra-observer variation (16). In the same study, thigh circumference measurements were made at an estimated half-way point and stifle position was not taken into account during measurements. These factors may have decreased consistency. It is important to use a flexible tape which easily follows the contours of the limb, yet does not stretch. It is also critical to use strategies to minimize the differences in the amount of tension placed on the tape during serial measurements. A tape measure with a spring loaded tension gauge may also help to reduce variation between observers and is a reason why we used the Gulick II.

It is clear that thigh girth decreases distally along the femur. Muscles of the thigh are quite prominent proximally, and decrease in size distally. In particular, as the quadriceps muscles approach the stifle joint, they become musculotendinous structures, and finally become the patellar tendon near the stifle joint. We also found it technically easier to perform measurements at the 70% thigh length as compared with 50% because this site is distal to the flank fold.

Although there were no statistically significant differences in measurements before and after clipping hair, the average difference between clipped and unclipped measurements was 3 and 7 mm at the 70 and 50% thigh measurement locations, respectively. However, the difference may have been greater in dogs with longer hair than the short-haired hound dogs used in this study. Unclipped vs. clipped hair measurements were only made with the stifle at an estimated standing angle because of time constraints immediately prior to surgery. Despite the lack of statistically significant differences, we recommend that thigh girth be measured as consistently as possible, and that all serial measurements on an individual patient be made with the hair clipped short for ease of measurement if possible.

The degree of stifle flexion or extension may significantly affect measurement of thigh girth. Thigh girth measurements with the stifle fully extended were consistently less than those made with the stifle in a standing position or with the stifle flexed. Although there were no differences in the measurements made with the stifle in a flexed or standing position, we found that measurements with the stifle flexed are technically more difficult and require close attention to placing the measuring tape so that the tape does not slip down the leg. Placement of the limb in an estimated standing position requires knowledge of the standing angle of the joints in that particular patient with regards to breed, size and conformation. Estimated standing positions are somewhat subjective in a recumbent patient, however, fully extended positions may be more consistent. Placing the hind limb in an extended stifle position may have less variability and result in more consistent measurements of thigh girth. Measurements made in standing dogs in one study resulted in low agreement among investigators and may be due to the variations in the standing stifle angle and differences in muscle tension while standing (8). There is evidence from previous studies that maintaining the stifle in a standing position may result in unacceptable variability (8, 13). Subjectively, it seemed easier to measure thigh girth with the stifle fully extended by a handler, and with the dogs in lateral recumbency in our study.

Sedation resulted in a non-significant decrease in thigh girth. The differences were greatest at the 50% thigh length measurement, presumably because muscle mass is greater proximally and any changes in muscle tone would likely be accentuated in regions of greater muscle mass. Measurements at the 70% thigh length with the limb in either an extended or standing position differed by only 1 mm, on average, with and without sedation. The dogs used in this study were generally calm and tolerated restraint in lateral recumbency very well, similar to most, but not all, clinical patients. Sedation did not significantly affect measurements, suggesting that if the dog is reasonably calm, the additional relaxation provided by acepromazine did not appreciably affect the measurements. In a clinical setting, sedation may be necessary in certain situations, such as for an excited patient or to obtain radiographs, and thigh circumference measurements should be ideally obtained using a technique that is minimally affected by sedation. Acepromazine was the only sedative used in the study and other medications may produce different results (i.e., alpha-two agonist or benzodiazepines). However, our results suggest that if a dog is reasonably calm, sedation has minimal contribution to thigh circumference measurements. Although we cannot speculate how thigh circumference is affected by a dog that struggles and has tense muscles, our results suggest that thigh circumference measurements with the stifle in an extended position are similar in relaxed dogs and in dogs following sedation with acepromazine, and it is possible that measurements of tense dogs following sedation may give results that more closely reflect those obtained if the dogs were relaxed.

There was a significant decrease in thigh girth at both thigh length points for all limb positions 2 weeks after surgery. The changes in thigh girth are presumably due to muscle atrophy following surgery and are consistent with human studies measuring muscle mass post-surgery (1, 2). In this study, dogs did not use their affected limbs to an appreciable extent during the 2 weeks study period after surgery. Percent changes in thigh girth were similar at both limb length sites, although the absolute amount of change was greater at the 50% thigh length site, suggesting that areas with the greatest amount of muscle mass are affected the most by muscle atrophy which has also been shown in human anterior cruciate ligament injuries (1). Although the limbs were not reclipped 2 weeks after surgery, the amount of hair regrowth was minimal during that time, and the magnitude of changes in thigh girth following surgery was much greater than that following clipping if the measurements are made with the stifle held in a consistent position and at a consistent thigh length. This suggests that the measuring techniques used in this study are sensitive enough to determine changes in thigh girth within 2 weeks of an event resulting in minimal weight-bearing. Future studies are necessary to examine the relationship between thigh girth and actual muscle mass to establish whether thigh girth is a reasonable method of assessing changes in muscle mass following injury and rehabilitation.

It is desirable to have a technique for measuring thigh girth that is repeatable between different observers so that reliable results may be obtained regardless of the observer. In this study, both investigators practiced measurements prior to the study start. With proper training and attention to detail, differences between investigators were relatively small. The difference between observers was 3.5% or less for each category assessed. The greatest differences were seen with the limb in the flexed and standing positions, suggesting that it was more difficult to obtain consistent measurements. Although the agreement between observers was slightly greater at the 50% thigh site, both observers subjectively felt that it was technically more difficult to properly obtain measurements at this site because of the presence of the flank skin in some dogs.

The authors performed the ICC to evaluate the repeatability of measuring thigh circumference. The ICC correlations indicated excellent agreement for both inter- and intra-observer variability (Table 4). Smith et al. found significant inter- and intra-observer variability when measuring thigh circumference midway between the hip and stifle in Labrador Retrievers (13). Similar findings were found by Bascuñán et al. in a two phase cadaveric and clinical study measuring thigh circumference at the 50% TL (8). This contrasts with our findings, where there was significant reliability between observers following training at both the 50 and 70% TL in the extended stifle position groups. We suspect that although TL location was taken into consideration for these previous studies, the degree of extension or flexion of the stifle joint may significantly affect thigh girth measurements. In addition, the thigh length measurements were made between two very distinct landmarks, the tip of the greater trochanter and the distal aspect of the lateral fabella. Others have measured from the greater trochanter to the lateral femoral condyle, which is less precise. We believe that less variation in measurement location should improve repeatability. While we acknowledge that marking the thigh length location would likely influence the repeatability of the triplicate measurements, measurement of the thigh length as described is quite repeatable in skeletally mature dogs. In fact, the thigh length measurements were within 3 mm between the preoperative and 2 weeks postoperative times. Our experience in determining thigh length in clinical patients is similar regarding measurements over several months.

It is unknown how slight variations in measurement technique may affect thigh circumference in clinical practice and we recommend adherence to techniques described here to obtain the most accurate results. It is also unknown if the results found in the study here could be extrapolated to other situations, such as making measurements in a standing position. In fact, it is our clinical experience that measurements made in a standing position are variable, as suggested by Bascuñán et al. (8) Finally, our findings should be further validated in dogs of different sizes and body type.

## Conclusions

Determination of thigh girth may be useful as an outcome measure if appropriate measuring technique is used. Currently, we prefer performing measurements at the 70% thigh length, with the hair clipped, the limb held in an extended position and with the animal relaxed, but not necessarily sedated. Standardizing conditions can minimize intra- and inter-observer measurement differences between observations on an individual patient. It is also important that those performing thigh girth measurements practice their technique and compare results to assure consistency.

## Author contributions

All authors listed have made a substantial, direct, and intellectual contribution to the work, and approved it for publication.

### Conflict of interest statement

The authors declare that the research was conducted in the absence of any commercial or financial relationships that could be construed as a potential conflict of interest.

## Footnotes

^1^Country Technology, Inc., Gays Mills, WI

^2^Vet One, MWI Animal Health, Boise, ID
